# Prevalence and antibiotic resistance of the ESBL producing enterobacteria strains isolated in Bologhine hospital during the years 2007-2010

**DOI:** 10.1186/1753-6561-5-S6-P136

**Published:** 2011-06-29

**Authors:** W Amhis, I Boudjelti, N Sahel, L Benbedka, S Benmesbah, W Hamzi

**Affiliations:** 1 Laboratoire Central de Biologie, Hôpital Bologhine IBN Ziri Algiers, Algiers, Algeria

## Introduction / objectives

To determine the prevalence and the resistance of the extended-spectrum betalactamase (BLSE) producing enterobacteriacea strains in our hospital.

## Methods

All enterobacteriacea strains were screened prospectively between January 2007 and December 2010 for ESBL on the basis of a positive double disk synergy test or positive Ceftazidime and Cefotaxime clavulanic acid combination disks tests. To detect ESBL in the species producing Ampc Betalactamase a modified double disk diffusion test (MDDT) by Pitout et al. was used.

## Results

Among 979 strains isolated during this period, 10.82 % (106) were ESBL producers. These bacteria were isolated in 94.33% in the inpatients specimens. Their distribution was essentially in the ICU (22.64%),the surgery (20.75%) and the pediatrics 21.21%.They were more frequently isolated from urines (36.79%), pus (33%), peritoneal liquid (15,15%)and bacteremia (12,26%).This betalactamase was produced by Klebsiella pneumoniae in 60.37%, EÂ .coli in 21.69% Enterobacter cloacae in 15% . Klebsiella, EÂ .coli and Enterobacter cloacae were also resistant to Fluoroquinolone (28.12%, 34.78% and 31.25%), aminoglycoside (71.8%,56.52% and 50%) and Cotrimoxazole (28.12%,30.43% and 31.25%).

## Conclusion

This study showed that the ESBL producing enterobacteria strains rate is high in our hospital. Other strain than Klebsiella and E.coli are expressing ESBL .It is the case of Enterobacter cloacae . So we have to perform the detection of these strains in our laboratory, because these bacteria may function as a reservoir for plasmids carrying ESBL-encoding genes. In front of this situation we have to enhance the hygiene measures in the units were these strainsÂ were isolated to limit their diffusion.

## Disclosure of interest

None declared.

